# Optimal ablation index parameters for radiofrequency ablation therapy of atrial fibrillation

**DOI:** 10.12669/pjms.38.3.4971

**Published:** 2022

**Authors:** Xiaoru Qin, Xiaofei Jiang, Qiyan Yuan, Guangli Xu, Xianzhi He

**Affiliations:** 1 Xiaoru Qin, Department of Cardiology, Zhuhai People’s Hospital, Zhuhai Hospital Affiliated with Jinan University, 79 Kangning Road, Zhuhai, 519000 Guangdong Province, China; 2 Xiaofei Jiang, Department of Cardiology, Zhuhai People’s Hospital, Zhuhai Hospital Affiliated with Jinan University, 79 Kangning Road, Zhuhai, 519000 Guangdong Province, China; 3 Qiyan Yuan, Department of Cardiology, Zhuhai People’s Hospital, Zhuhai Hospital Affiliated with Jinan University, 79 Kangning Road, Zhuhai, 519000 Guangdong Province, China; 4 Guangli Xu, Department of Cardiology, Zhuhai People’s Hospital, Zhuhai Hospital Affiliated with Jinan University, 79 Kangning Road, Zhuhai, 519000 Guangdong Province, China; 5 Xianzhi He, Department of Cardiology, Zhuhai People’s Hospital, Zhuhai Hospital Affiliated with Jinan University, 79 Kangning Road, Zhuhai, 519000 Guangdong Province, China

**Keywords:** Atrial fibrillation, Ablation index, Catheter ablation

## Abstract

**Objectives:**

To explore the optimal ablation index (AI) parameters for radiofrequency catheter ablation (RA) for treating atrial fibrillation (AF).

**Methods:**

Patients with AF (186) who underwent bilateral PVAI in the Department of Cardiology, Zhuhai People’s Hospital, Guangdong Province, from March 2018 to October 2019 and received catheter ablation as first-round treatment, were grouped according to the received AI. Control group included patients (95) who received the recommended AI ablation (350–400 for posterior wall, 400–450 for non-posterior wall). Patients in optimal AI group were ablated with optimal AI (300–330 for posterior wall, 350–380 for non-posterior wall). Recurrence was defined as any AF, atrial tachycardia, or atrial flutter lasting more than 30 seconds without anti-arrhythmic drugs after the 3-month blank period.

**Results:**

Of 186 patients, 66 patients had paroxysmal atrial fibrillation and a mean CHA_2_DS_2_-VASc score of 2.83±1.64. Isolation rates of bilateral PVI in both groups were 91.4% and 93.6%, for patients with paroxysmal atrial fibrillation, and 81.7% and 80% for patients with persistent atrial fibrillation (P > 0.05). Left atrial function index (LAFI) decreased under the condition of sinus rhythm at the 3rd and 6th months (P < 0.05). LAFI improvement was significantly better in the optimal AI group than in the control group (P < 0.05). Rates of pain and cough during the ablation, and postoperative gastrointestinal discomfort and use of PPIs were higher in the control group (P < 0.05). The recurrence rate was 14.7% and 14.3% after 12 months of follow-up, respectively, and the difference was not statistically significant (P > 0.05).

**Conclusion:**

Radiofrequency ablation of AF, guided by optimal AI combined with impedance, can minimize atrial injury, prevent atrial failure, promote the recovery of atrial function, reduces intraoperative cough, pain, and postoperative gastrointestinal discomfort and use of PPIs.

## INTRODUCTION

Radiofrequency ablation (RA) to cure atrial fibrillation (AF) has become increasingly prevalent recently. However, many patients suffer from atrial dysfunction and cardiac insufficiency due to AF, which may lead to the enlargement of the left atrium or the left and right atria, and potentially to left atrial failure.^1^ Radiofrequency (RF) catheter ablation therapy to achieve electrical isolation of the pulmonary veins (PVs), is the cornerstone of treatment for AF.^2^ In recent years, studies have introduced ablation index (AI), a lesion quality marker that combines contact force, pressure and time. During RF ablation guided by the three-dimensional CARTO system, the degree of damage at the ablation points can be evaluated in real time, to ensure the transmural damage at each ablation point,^3^ potentially reducing operation-related complications.^4^,^5^ Due to its improved VisiTag points and AI module, the use of ST-SF catheter is associated with higher the success rates of the operation and reduced operation-related complications. However, higher AI values and numbers of ablation points may increase injury of the left atrium and surrounding organs, affect atrial function, and lead to complications such as atrial failure and esophagoatrial fistula.^6^,^7^ In this study, we used the AI as a reference and adjusted AI parameters in combination with impedance changes to guide AF ablation. We grouped the results according to the different AI values and monitored the effects of PV isolation and sinus rhythm maintenance, to assess the changes in atrial function and overall clinical symptoms.

## METHODS

A total of 186 patients with paroxysmal or persistent AF who underwent bilateral PVAI in the Department of Cardiology, Zhuhai People’s Hospital, Guangdong Province, from March 2018 to October 2019 were enrolled in this retrospective study. RF catheter ablation was performed according to the type and the intraoperative condition of the AF. Partial persistent AF was treated with parietal line ablation, superior vena cava isolation or ectopic focus ablation of the pulmonary vein. All patients had indications for catheter ablation, received ablation treatment for the first time and had signed a written informed consent form for catheter ablation before the operation. Data on the age, sex, BMI, hypertension, diabetes, type of atrial fibrillation, left atrium diameter, CHA_2_DS_2_-VASc score, symptoms of cough and pain during the operation, single/double isolation rate of the pulmonary vein, VisiTag points, ablation impedance, postoperative gastrointestinal discomfort, and PPIs were available for all included patients.

### Ethics Approval

Study was approved by the ethics committee of the Zhuhai People’s Hospital (Ref. LW-2021-06).

### Preoperative preparation

Medical history recording, physical examination and the following tests were performed: routine blood, routine urine, routine fecal, blood coagulation, liver function, renal function, electrolytes, blood lipids, and free thyroid function, electrocardiogram, chest X-ray, and transthoracic and transesophageal echocardiography. CHA_2_DS_2_-VASc and HAS-BLED scores were calculated. Antiarrhythmic drugs were discontinued for -five half-lives (except amiodarone) and anticoagulant therapy was given before the operation. Warfarin treatment was not stopped, while patients receiving non-vitamin K antagonist oral anticoagulants (rivaroxaban or darbicarboxine) were required to switch to low-molecular-weight heparin after admission. All patients stopped anticoagulant therapy the morning of the operation.

### Cardiac color Doppler ultrasonography

Real-time three-dimensional echocardiography (RT-3DE) measurements were carried out. Quantitative analysis was performed using Qlab software. In the 3DQA mode, clear sections of the left atrium were selected from the 3D database and placed on three mutually orthogonal reference planes. The selected phase automatically depicted the endocardial surface of the left atrium and mapped the volume. Left atrial maximum volume (LAVmax) was measured at the end of the systolic period, left atrial presystolic volume (LAVp) was measured at the beginning of the atrial systolic period (start of the P wave), and left atrial minimum volume (LAVmin) was measured at the end of the diastolic period. Left atrial emptying fraction LAEF = (LAVmax – LAVmin) / LAVmax, left atrial passive emptying fraction LAPEF = ((LAVmax – LAVp) / LAVmax, and left atrial active emptying fraction LAAEF = (LAVp – LAVmin) / LAVp were calculated. Left atrial function index was calculated as follows: body surface area (BSA) was calculated according to the height and weight of the subject. Maximal left atrial volume index (LAESVI) after BSA correction was established using the equation: LAESVI = LAVmax / BSA. Left atrial function index was calculated as LAFI = (LAEF × LVOT-VTI) / LAESVI, where LVOT-VTI is the left ventricular outflow tract velocity-time integral.

### Ablation procedure

Using local anesthesia, the left femoral vein was punctured, and a spatially adjustable 10-pole electrode was placed into the coronary sinus (CS). The right femoral vein was punctured and two 8.5F long sheaths were inserted. The atrium septum was punctured under the guidance of the CS internal electrode and X-ray fluoroscopy. Heparin (100 IU/kg) was given after completion, and the PentaRay star mapping electrode was directed to the left atrium along the long sheath. Under the guidance of the Carto3 electrophysiological navigation system (Carto3, Version 4, Biosense Webster, Johnson & Johnson, Irvine, California, USA), three-dimensional models of the left atrium and pulmonary vein were constructed by rapid anatomical modeling (Fast Anatomia Mapping, FAM) after respiratory compensation of the left inferior pulmonary vein. The ST-SF cold saline perfusion pressure monitoring catheter (Thermo Cool Smart Touch® SF, ST-SF) was directed to the left atrium and bilateral PVI was obtained based on AI reference impedance. Other appropriate procedures for catheter ablation were chosen for some patients with persistent AF according to the type of AF and the occurrence of AF during the operation. The power mode was set to 25–45 W. The flow rates of the saline infusion were two mL/minutes without ablation, eight mL/minutes at 25–30 W ablation, and 15mL/min at 31-45 W ablation. The ablation power was 25 W for the posterior wall and 30 W for the non-posterior wall. During the ablation process, VisiTag (Carto Visitagtm Module, Biosense Webster, Johnson & Johnson, Irvine, California, USA) parameters were set to quantify the stability of the catheter and the ablative tissue contact. The VisiTag point in the center settings were as follows: maximum displacement of the catheter standard deviation was 2.5 mm, minimum time for maintaining stability was -three seconds, and the pressure range was five~twenty g. VisiTag points were displayed with a radius of three mm. During the ablation process, two VisiTag points were connected to ensure that the distance between the two points was less than six mm. The ablation process started from the right upper pulmonary vein vestibule to reduce the vasovagal response. Point-by-point ablation was performed. The pulmonary vein potential recorded on the PentaRay electrode was lost and maintained for 30 minutes or more as the ablation endpoint (entrance block). Intravenous fentanyl and midazolam were used to relieve pain during the operation. Heart rhythm, blood pressure, and blood oxygen saturation were monitored, and single-loop isolation was recorded. Single-loop isolation was defined as the completion of PVI (including the interpulmonary crest) by point-to-point ablation of the catheter in a single direction along the preset ablation path.

### AI values of the control and optimal AI groups

Based on the received AI, patients were retrospectively divided into two groups, the control and the optimal AI. The patients who received AI values of 350–400 for the posterior wall, 400–450 for the non-posterior wall (values, determined based on the data from animal and clinical trials for the ST-SF catheter)^6^,^7^ were assigned into a control group. Patients who received AI values of 300–330 for the posterior wall, 350–380 for the non-posterior were included in the optimal AI group. Optimal ablation AI values were based on the anatomical parameters of the left atrial pulmonary vein vestibular chamber combined with the actual ablation effects and the patient’s pain and digestive tract reaction parameters, and further verified clinically, and adjusted by referring to the ablation impedance of each patient (110-140 Ω). The records of the changes in the left and right atria, left atrial function, and the incidence of severe perioperative complications were compared between the two groups at one, three, and six months after the operation.

### Postoperative management

Postoperative protocol for patients in the control group dry included fasting for six hours after surgery, and a PPI (rabeprazole) for one month following the procedure. Patients that received optimal AI group treatment were not required to fast, abstain from water, or take PPIs after surgery. All the patients were discharged from the hospital after 24-48 hour of routine observation, and given oral anticoagulants (rivaroxaban/darbicarboxine/warfarin) for two-three months as appropriate, based on the AF and the CHA_2_DS_2_-VASc score.

### Postoperative follow-up

All patients received new oral anticoagulant (rivaroxaban) or warfarin anticoagulant for two to three months postoperatively. For those taking warfarin, the international standard ratio was adjusted to 2-3. Antiarrhythmic drugs were not routinely taken after paroxysmal atrial fibrillation. Amiodarone or propafenone were prescribed two to three months later for patients with frequent attacks or persistent AF. If symptomatic bradycardia or heart rate was lower than 50 beats/min, antitemperament drugs were stopped or reduced in time. If AF occurred within three months of the gap period, electrical cardioversion was given. During the follow-up period, each patient was required to self-measure their pulse three times a day. Three months after the operation, 24 hour dynamic electrocardiogram was reviewed. Ambulatory electrocardiogram (24 hour ) was reexamined at six and 12 months postoperatively. Patient were contacted for telephone follow-up every 3 months. Patients with palpitation, chest tightness and other symptoms, received timely ECG or dynamic ECG examination in addition to pulse self-monitoring. Criteria for ablation success: no atrial arrhythmias lasting more than 30 seconds without the use of antiarrhythmic drugs after the three month gap period of catheter ablation.

Measured data that conformed to a normal distribution were expressed as the mean±SD, and counted data were expressed as rates. A P < 0.05 was considered statistically significant.

## RESULTS

Baseline characteristics of 186 patients with paroxysmal or persistent AF that underwent bilateral PVAI are shown in Table-I. All 186 patients received bilateral PVAI, with PentaRay electrode mapping potential as a reference. Of them, 54 patients (29%) with persistent AF underwent roof-line ablation, 19 patients (10.2%) with persistent AF underwent superior vena cava isolation, and 11 (5.9%) underwent ablation of the ectopic trigger foci outside the pulmonary vein. Twenty-six patients (14%) with typical atrial flutter underwent linear ablation of the tricuspid valve isthmus, and 112 patients received electro cardioversion. There were no significant differences in the single-loop isolation rates of pulmonary vein isolation, Visi-tag points, or ablation impedances between the two groups (P > 0.05; Table-II).

**Table I:** Baseline data of conventional AI and optimal AIs of patients with AF (n = 186; intergroup comparison P > 0.05).

Parameter	Conventional AI (95 cases)	Optimal AI (91 cases)
Age (years, Mean±SD)	65±11.76	66±12.32
Male (example %)	52(54.7)	51(56.0)
BMI (kg/m2, Mean±SD)	25.69±3.64	26.25±3.51
Hypertension (case %)	58(61.1)	54(59.3)
Diabetes mellitus (case %)	28(29.5)	25(27.5)
Paroxysmal AF	35(36.8)	31(34.1)
persistent AF	60(63.2)	60(65.9)
LAD (mm, Mean±SD)	42.35±5.48	43.18±5.62
CHA_2_DS_2_-VASC	2.86±1.68	2.73±1.53

**Table II:** Single-loop isolation rates of pulmonary vein isolation (P > 0.05 for inter-group comparison).

Parameter	Conventional AI	Optimal AI
Paroxysmal AF (case)	35	31
Biring isolation rate (%)	32(91.4)	29(93.6)
Right ring isolation rate (%)	33(94.3)	29(93.6)
Left ring isolation rate (%)	33(94.3)	29(93.6)
Persistent AF	60	60
Biring isolation rate (%)	49(81.7)	48(80.0)
Right ring isolation rate (%))	51(85.0)	51(85.0)
Left ring isolation rate (%)	51(85.0)	51(85.0)
Visitag point	114.5±11.68	115.6±11.36
Ablation impedance (Ω)	129.6±5.36	128.8±5.28

Control group had more cough, pain, postoperative gastrointestinal discomfort symptoms and postoperative use of PPIs compared to the optimal AI group (Table-III).

**Table III:** Symptoms during and after the operation and the use of PPIs after the operation (P< 0.05 for inter-group comparison).

Parameter	Conventional AI(95 cases)	Optimal AI (91 cases)
***During operation:***		
Cough	19(20%)	7(7.7%)
Pain	78(82.1%)	23(25.3%)
***After operation:***		
Pain	26(27.4%)	0(0%)
The use of PPIs	84(88.4%)	2(2.2%)

Changes in the left and right atrium color Doppler ultrasound and left atrial function before and after RA in the two groups

Of 186 patients with AF, 37 (19.9%) had paroxysmal AF with left and right atria of normal size before the operation, 98 (52.7%) had paroxysmal or persistent AF with an enlarged left atrium, and 51 (27.4%) had persistent AF with enlarged left and right atria. In patients with AF with an enlarged left atrium, the size of the atrium started decreasing one to three months after RA. In 16 cases (16.3%) left atrium returned to its normal size after six months. In patients with AF with enlarged left and right atria, right atrium returned to normal size three to six months after RA, while the size of the left atrium decreased but did not return to normal. After RA, the left atrium of three patients with AF with a normal left atrium size showed a tendency to expand and the function of the left atrium decreased (Table-IV).

**Table IV:** Left atrial function index, left atrial diameter, volume index, and evacuation fraction of three patients with a normal left atrium before and after RA.

Detection time	LAFI	LAD(mm)	LAVmax(ml)	LAVmin(ml)	LAVp(ml)	LAPEF(%)	LAAEF(%)	LAEF(%)
***The first case***								
Preoperative	0.53	31	36	17	27	0.26	0.38	0.52
1 month after ablation	0.50	32	37	17	28	0.25	0.37	0.51
3 month after ablation	0.49	33	38	17	29	0.25	0.37	0.51
6 month after ablation	0.45	34	40	19	30	0.24	0.36	0.50

LAFI started to increase one month after the procedure, and that increase was statistically significant at three and six months after the operation (P < 0.05). Changes in the optimal AI group were more significant, as compared to the control group (P < 0.05). There was no significant difference in LAD size at one month after surgery (P > 0.05) in the control group, and LAD at three and six months after surgery had decreased compared with the preoperative group (P < 0.05). In contrast, LAD size in the optimal AI group was significantly higher than that of the control group (P < 0.05). There was no significant difference in left atrial volume index 1 month after the operation (P > 0.05). LAVmax, LAVp, and LAVmin decreased at three and six months after the operation in the control group. These changes were more obvious and statistically significant in the optimal AI group (P < 0.05). The increase in LAAEF and LAEF at one, three and six months after the operation was significantly higher in the optimal AI group, as compared to the control group (P < 0.05). There was a significant difference in LAPEF in the optimal AI group at three and six months after the operation (P < 0.05) (Table-V).

**Table V:** Comparison between the two groups of the left atrial function indexes, left atrial diameters, volume indexes, and emptying fractions before and after radiofrequency catheter ablation.

Detection time	LAFI	LAD (mm)	LAVmax (ml)	LAVmin (ml)	LAVp (ml)	LAPEF (%)	LAAEF (%)	LAEF (%)
***Conventional AI:***								
Preoperative	0.39±0.17	39.5±3.84	45.56±14.02	25.9±11.31	36.5±13.56	0.21±0.08	0.30±0.09	0.46±0.07
1 month after surgery	0.40±0.18	39.1±3.68	45.28±13.61	25.6±11.05	36.5±12.68	0.21±0.07	0.32±0.11	0.46±0.08
3 month after surgery	0.48±0.17	38.6±3.23	43.47±12.75	22.86±9.85	33.8±12.53	0.22±0.07	0.34±0.08	0.48±0.06
6 month after surgery	0.52±0.23	37.3±2.89	41.18±11.31	19.96±7.65	30.56±9.5	0.23±0.05	0.36±0.07	0.49±0.05
***Optimal AI:***								
Preoperative	0.38±0.15	39.4±3.82	45.54±14.12	25.9±11.31	36.5±13.56	0.21±0.08	0.30±0.09	0.46±0.07
1 month after surgery	0.43±0.18	38.6±3.28	45.18±13.31	24.6±10.85	36.0±12.67	0.21±0.07	0.33±0.11	0.47±0.08
3 month after surgery	0.51±0.17	37.6±3.13	41.77±12.65	21.46±9.85	32.2±12.23	0.23±0.07	0.34±0.08	0.49±0.06
6 month after surgery	0.62±0.23	36.2±2.79	39.08±10.31	18.36±6.65	29.56±9.5	0.24±0.05	0.38±0.07	0.51±0.05

***Note:*** In the control, there were significant differences at 3 and 6 months after the operation compared with the values before the operation (P < 0.05). The differences between the optimal AI and the control at 3 and 6 months after surgery were statistically significant (P < 0.05). LAFI: left atrial function index; LAD: left atrial anteroposterior diameter; LAVmax: left atrial maximum volume; LAVmin: left atrial minimum volume; LAVp: left atrial presystolic volume; LAPEF: left atrial passive emptying fraction; LAAEF: left atrial active emptying fraction; LAEF: left atrial emptying fraction.

After 12 months of follow-up, recurrence occurred in 14 patients (14/95, 14.7%) in the control group and 13 (13/91, 14.3%) in the best AI group. There was no statistically significant difference between the two.

## DISCUSSION

This retrospective study compared changes in the left and right atria, left atrial function, and the incidence of severe perioperative complications between the two groups of patients who were treated with radiofrequency ablation different AI values, and showed that adjusted AI parameters in combination with impedance changes reduced symptoms of discomfort, and the need for PPIs, as well as improved the recovery of the left atrium function.

Radiofrequency ablation (RA) is the main treatment method for restoring and maintaining sinus rhythm in patients with paroxysmal and persistent atrial fibrillation.^8^ Preclinical work has demonstrated that factors, such as ablation duration, tissue contact force (CF), power, and catheter irrigation are determinants of radiofrequency lesion size.^9^-^11^ RA, performed under the guidance of AI, can significantly increase the overall success rate of the operation, shorten the operation time, and is not associated with increased incidence of complications.^4^,^5^

In this study, the PentaRay star mapping electrode FAM was used to accurately position the pulmonary vein atrium and set VisiTag parameters, and a 56-hole cold saline perfusion pressure monitoring ablation catheter was used. There was no significant difference in the single cycle isolation rate between the two study groups. In the control group, there were more symptoms of discomfort and greater use of PPIs in intraoperative and postoperative period, the recovery of left atrial function was slow, and some patients had excessive ablation. In patients in the optimal AI group, the symptoms of discomfort, and the need for PPIs were significantly reduced. Our results suggest that the function of the left atrium recovered better, probably due to avoiding an ablation effect due to insufficient ablation.

Numerous studies in animal models as well as several multicenter clinical trials attempted to evaluate thickness of the left atrial wall, as well as the optimal AI for blocking pulmonary venous potential conduction on the anterior wall/top and the posterior wall/bottom of the left atrium.^12^-^17^ More clinical studies are required to determine whether the AI reference value reported are the best ablation parameter for pulmonary vein isolation, and whether there is excessive ablation. A higher AI value will increase the duration of the operation and the probability of adjacent tissue injury (esophageal and lung tissue, etc.), and increase the probability of serious complications.^18^,^19^ Current high-power ablation can contribute to complete isolation of pulmonary veins by ensuring better continuity between adjacent lesions, while reduced lesion depth can still achieve transmural penetration in atrial tissues, further supporting the successful results reported in this study.^20^

RF catheter ablation employs thermal damage that depends on impedance, output power, adhesion pressure, and ablation time.^9^-^11^ When RA is performed with the same basic impedance, the value of AI positively correlates with the extent of injury. In contrast, when RA is performed with the same AI target value, there is a negative correlation between the basic impedance and the damage range. RA with low impedance is associated with higher risk of steam burst and / or perforation complications. In our study, we demonstrated that the basic impedance of a patient did not fluctuate significantly with time, and AI positively correlated with the extent of injury. However, basic impedance varied greatly among different patients, and the target value of AI, therefore, needed to be personalized according to the basic impedance. For individuals with high basic impedance, the desired effect can be achieved by increasing the AI value, and vice versa.^21^,^22^ During the ablation, the basic impedance in our study was controlled at 110–140 Ω in combination with the appropriate AI to maximize the ablation range and minimize the risk of complications. We know that power is the main factor causing thermal injury, and time is an important condition of heat conduction, so higher power ablation mainly causes impedance heat injury rather than heat conduction injury, and its damage volume is characterized by a large diameter and shallow depth.^23^ The results of this study will allow clinicians to develop treatment protocols to further improve long-term outcomes for patients with paroxysmal and persistent atrial fibrillation undergoing RA.

### Limitation of the study

The main limitation of this study is that it was a single-center retrospective study that relied upon accurate, detailed and available patient data. The study cohort was of relatively small size, and not randomized. Further large-scale, multicenter, prospective studies are needed.

## CONCLUSION

Radiofrequency ablation of AF, guided by optimal AI combined with impedance, can effectively minimize atrial injury, prevent atrial failure, promotes the recovery of atrial function, and reduces intraoperative and postoperative discomfort and use of PPIs. Further large-scale, multicenter, prospective studies are needed to provide improved long-term outcomes for patients with paroxysmal and persistent atrial fibrillation undergoing RA.

### Authors’ contributions:

**XQ:** Designed the project.

**XJ, QY & YG:** Were involved in data collection and data analysis.

**XQ:** Prepared the manuscript; XH edited the manuscript.

**XQ:** Is responsible and accountable for the accuracy or integrity of the work.

All authors read and approved the final manuscript.
